# (Pro)renin receptor promotes colorectal cancer through the Wnt/beta-catenin signalling pathway despite constitutive pathway component mutations

**DOI:** 10.1038/s41416-018-0350-0

**Published:** 2018-12-17

**Authors:** Juan Wang, Yuki Shibayama, Anqi Zhang, Hiroyuki Ohsaki, Eisuke Asano, Yasuyuki Suzuki, Yoshio Kushida, Hideki Kobara, Tsutomu Masaki, Zhiyu Wang, Akira Nishiyama

**Affiliations:** 1 Department of Medical Oncology, The Second Affiliated Hospital of Zhejiang University School of Medicine, Hangzhou, 310009 Zhejiang Province China; 2 Department of Pharmacology, Faculty of Medicine, Kagawa University, Kagawa, 761-0793 Japan; 3 Department of Immuno-oncology, Fourth Affiliated Hospital of Hebei Medical University, Shijiazhuang, 050000 Hebei Province China; 4 Department of Medical Biophysics, Kobe University Graduate School of Health Sciences, Kobe, 650-0017 Japan; 5 Department of Gastroenterological Surgery, Faculty of Medicine, Kagawa University, Kagawa, 761-0793 Japan; 6 Department of Diagnostic Pathology, Faculty of Medicine, Kagawa University, Kagawa, 761-0793 Japan; 7 Department of Gastroenterology and Neurology, Faculty of Medicine, Kagawa University, Kagawa, 761-0793 Japan

**Keywords:** Colorectal cancer, Cancer therapy

## Abstract

**Background:**

Although constitutive activating mutations in the Wnt/β-catenin signalling pathway are important for colorectal cancer development, canonical signalling through Wnt ligands is essential for β-catenin activation. Here, we investigated the role of (pro)renin receptor ((P)RR), a component of the Wnt receptor complex, in the pathogenesis of colorectal cancer.

**Methods:**

(P)RR silencing was performed in human colorectal cancer cells containing constitutive activating mutations in the Wnt/β-catenin pathway. (P)RR overexpression was induced in normal colon epithelial cells. Protein and mRNA levels of pathway components were detected, and Wnt signalling activity was measured using a β-catenin reporter. Cell proliferative activity and apoptosis were evaluated using WST-1 assay and flow cytometry. Xenografts were induced in nude mice.

**Results:**

(P)RR expression was greater in colorectal cancer tissues and cells than in normal colorectal samples. Patients with strong (P)RR expression took more proportion in groups with poorly-differentiated, advanced and rapidly-progressing cancers. (P)RR silencing attenuated the pathway in colorectal cancer cells, impaired their proliferation in vitro and vivo. (P)RR overexpression enhanced the pathway and proliferation of normal colon epithelial cells.

**Conclusions:**

Aberrant (P)RR expression promotes colorectal cancer through the Wnt/β-catenin signalling pathway despite constitutive pathway-activating mutations. (P)RR is a potential diagnostic and therapeutic target for colorectal cancer.

## Introduction

Colorectal cancer (CRC) is the third most common cancer in males and the second in females^1^ and is the third leading cause of cancer-related deaths worldwide.^2^ Over one million newly diagnosed cases are reported annually and the disease-specific mortality rate is nearly 33%.^3^ Only 39% of CRC patients are diagnosed in the early stage, whose 5-year survival rate is 92%; furthermore, the 5-year survival rates of patients with regional and distant metastasis sharply decline to 53 and 11%.^4^ Therefore, the identification of effective diagnostic and prognostic indicators and therapeutic targets for CRC remains urgently needed.

Wnt/β-catenin signalling pathway plays pivotal roles in many biological processes, such as embryonic development, tissue homoeostasis and carcinogenesis.^5^ Excessive activation of the Wnt/β-catenin signalling induces aberrant cell proliferation and inhibits apoptosis,^6^ which contributes to genesis and progression of several types of human cancers including colorectal,^7^ gastric,^8^ prostate,^9^ breast^10^ and adrenocortical^11^ cancers. Wnt ligands are responsible for the activation of Wnt/β-catenin signalling and more than 19 secreted family members have been discovered.^12^ In the absence of Wnts, β-catenin degradation is constantly mediated by the destruction complex, which consists of the adenomatous polyposis coli protein (APC) and several other proteins. Binding of Wnts to the Wnt receptor complex, which includes Frizzled (Fzd) and low-density lipoprotein receptor related protein 6 (LRP6), induces LRP6 phosphorylation, disturbs the destruction complex and thus inhibits β-catenin degradation. Active β-catenin then translocates into the nucleus and binds with T-cell factor/lymphoid enhancer factor (TCF/LEF) to activate the expression of target oncogenes such as *CCND1* (encodes Cyclin D1)and *c-Myc*.^13–15^ Over 80% of colorectal tumours carry loss-of-function mutation in APC and approximately 5% carry activating mutation in β-catenin,^16,17^ which leads to the constitutive activation of the Wnt/β-catenin pathway and thus contributes to cancer development.^18^ However, recent studies have shown that Wnt3 silencing remarkably attenuated the activation of the Wnt/β-catenin pathway and proliferation of CRC cells with mutation in APC or β-catenin.^19^ These data indicate that Wnt ligands can further activate a mutated Wnt/β-catenin pathway in which signalling activity is constitutively active.

(Pro)renin receptor ((P)RR), a 350-amino acid protein encoded by an X chromosome-located gene, was initially discovered as an essential component in the renin-angiotensin system and ubiquitously expressed in the human body.^20^ However, Cruciat et al.^21^ demonstrated that (P)RR also acts as an adaptor protein that co-locates with the Wnt receptor complex and thus contributes to the activation of Wnt/β-catenin signalling, independent of the renin-angiotensin system. Recently, accumulating evidence has revealed that the expression of (P)RR is remarkably elevated in various human cancers, such as breast carcinoma,^22^ pancreatic ductal adenocarcinoma,^23^ glioma,^24^ and aldosterone-producing adenoma.^25^ In particular, we have recently demonstrated that aberrant (P)RR expression contributes to the genesis and progression of pancreatic ductal adenocarcinoma^23^ and glioma^24^ through the Wnt/β-catenin pathway.

Based on these findings, the present study was conducted to investigate the role of (P)RR in the pathogenesis of CRC. Our data suggest that (P)RR promotes CRC through Wnt/β-catenin pathway despite the presence of constitutive mutations in the pathway components. These findings may indicate (P)RR as a novel diagnostic and therapeutic target of CRC.

## Materials and methods

### Patients and their tissue samples

This study was conducted in accordance with the Declaration of Helsinki. All the protocols were approved by the Ethics Committee of Kagawa University. Informed consents were obtained from all the patients or their guardians. Colon tissue samples were collected from 60 patients diagnosed with CRC by histopathological examination. These patients underwent surgical resection from January 2008 to October 2016 and had their tissue sections kept in the Kagawa University Hospital. The 60 patients were composed of 33 males and 27 females and their ages ranged from 44 to 87 years old (average: 68 years old). Detailed clinicopathologic characteristics of the patients are shown in Supplementary Table S1. CRC stages were newly evaluated according to the 8th edition of TNM Classification of Malignant Tumours released by the Union for International Cancer Control (UICC). All the tissue samples included both cancer lesions and the matched adjacent normal tissues.

### Immunohistochemistry (IHC)

IHC was prepared and performed as previously described.^23,26^ Samples were respectively incubated with anti-(P)RR antibody^27,28^ (1: 3000), anti-active β-catenin antibody (1: 1000; Cell Signaling Technology, Danvers, MA, USA, catalog #8814), or anti-c-Myc antibody (1: 50; Abcam, Eugene, OR, USA, catalog #ab39688), as primary antibody for 1 h. Staining was considered positive when brown granules were observed in cells. Rabbit serum from which the (P)RR antibody was isolated served as the negative control to stain colorectal tissues using the same protocol as the (P)RR antibody. As previously described,^29,30^ the intensity of positive staining in cancer lesions was graded from 1 to 4 (1 as the weakest intensity and 4 as the strongest intensity) by four individuals, independently. The protein expression level in each cancer lesion was determined based on the average intensity score: 1 ≤ weak < 2, 2 ≤ middle ≤ 3, and 3 < strong ≤ 4.

### Cell culture

Two human CRC cell lines, DLD-1 and HCT116, as well as a normal colon epithelial cell line, CCD841 CoN, were purchased from the American Type Culture Collection (ATCC, Manassas, VA, USA). According to company’s instructions, DLD-1, HCT116 and CCD841 CoN cells were cultured in the RPMI-1640 medium (Sigma-Aldrich, St. Louis, MO, USA, catalog #R8758), McCoy’s 5a Modified Medium (Life Technologies, Carlsbad, CA, USA, catalog #16600-082) and Eagle’s minimum essential medium (ATCC, catalog #30-2003), respectively. Cells were maintained at 37 °C in a humidified incubator with 5% CO_2_/95% air.

### (P)RR silencing in CRC cells

Stealth small interfering RNA (siRNA) against (P)RR ((P)RR siRNA) (Life Technologies, catalog # HSS115476)^23^ and Stealth negative control siRNA (scrambled siRNA) (Life Technologies, catalog #12935-400) were used. CRC cells were transfected with siRNA using Lipofectamine RNAiMAX (Life Technologies, catalog #13778030) according to the manufacturer’s instructions. Cells were then maintained in serum-free conditions without antibiotics for 1 to 3 days until they were used for subsequent experiments. To induce stable lasting (P)RR silencing, cells were infected with a retroviral vector containing a short-hairpin RNA (shRNA) against (P)RR ((P)RR shRNA) (Origene Technologies, Rockville, MD, USA, catalog #TR314574) or a scrambled shRNA construct (Origene Technologies, catalog #TR30012) with Polybrene (Sigma-Aldrich, catalog #107689) in accordance with the manufacturers’ instructions. Infected cells were selected by 1 μg/mL puromycin. Efficiency of (P)RR silencing was confirmed by western blotting.

### Induction of (P)RR overexpression in normal colon epithelial cells

CCD841 CoN cells were transfected with pENTR4 plasmid vector (Invitrogen, Carlsbad, CA, USA, catalog #17424) containing (P)RR encoding gene *ATP6AP2*^24^ using Lipofectamine 3000 (Life Technologies, catalog #L3000001) according to the manufacturers’ instructions. Empty vector served as negative control.

### Real-time polymerase chain reaction (PCR)

Real-time PCR was performed as previously described^31^ using specific primer sets for the following human genes: *ATP6AP2* (encodes (P)RR) (forward: ggcgttggtggcgggtgtyy; reverse: agcccatggacaatgcagccac), *Wnt3* (forward: acttttgtgagcccaaccca; reverse: ttctccgtcctcgtgttgtg), *LRP6* (forward: aaacagacgggacttgcgat; reverse: aaacacaaagtccaccgcag), *CCND1* (forward: agctgtgcatctacaccgac; reverse: gaaatcgtgcggggtcattg), c-Myc (forward: ggacccgcttctctgaaagg; reverse: gtggacttcggtgcttacct) and 18S rRNA (forward: ggccctgtaattggaatgagtc; reverse: ccaagatccaactacgagctt). Human 18S rRNA served as the internal control.

### Western blotting

Western blotting analysis was performed as previously described.^23^ Loading amount of the whole cell extract protein for each sample was 50 μg. The primary antibodies used were anti-(P)RR antibody (1:1000),^27,28^ anti-Wnt3 antibody (1: 500; Abcam, catalog #ab32249), anti-total LRP6 antibody (1:800; Cell Signaling Technology, catalog #3395), anti-phosphorylated LRP6 antibody (Ser 1490, 1:800; Cell Signaling Technology, catalog #2568), anti-active β-catenin antibody (1:1000; Cell Signaling Technology, catalog #8814), anti-Cyclin D1 antibody (1:1000; Cell Signaling Technology, catalog #2922), anti-c-Myc antibody (1:1000; Cell Signaling Technology, catalog #5605) and anti-β-actin antibody (1:1000; Sigma-Aldrich, catalog #A5441). β-Actin served as internal control. Consistent results were obtained from at least three times of independent experiment.

### Immunoprecipitation (IP)

IP was performed as previously described.^31^

### Measurement of Wnt/β-catenin signalling activity

Wnt/β-catenin signalling activity was measured using a β-catenin firefly luciferase reporter plasmid containing TCF/LEF binding sites (TOP flash) (Addgene, Cambridge, MA, USA, Catalog #12456). The β-catenin reporter plasmid containing mutated TCF/LEF binding sites (FOP flash) (Addgene, Catalog #12457) served as negative control. As described previously,^32^ cells were transfected with β-catenin reporter plasmid and pRL-TK renilla luciferase reporter vector (Promega, Fitchburg, WI, USA, catalog #E2241), as an internal control for transfection efficiency, using the Lipofectamine LTX reagent (Life Technologies, catalog #15338030). Simultaneously, siRNA and pENTR4 plasmid vector were respectively transfected into CRC cells and CCD841 CoN cells. After transfection, cells were maintained in serum-free conditions for 2 to 3 days until they were examined using a Dual-luciferase Reporter Assay system kit (Promega, catalog #E1910) and a microplate reader (Corona Electric, Lethbridge, Canada, catalog #SH-9000), following the manufacturers’ instructions. Reporter activities were reflected by the luminescence intensity. Data were normalised by the value of pRL-TK renilla luciferase activity.

### Water-soluble tetrazolium salt (WST)-1 assay

To measure cell proliferative activity, WST-1 assay was performed as previously described.^23^

### Cell counting assay

Cell counting assay was performed as previously described.^23^

### Flow cytometry

Flow cytometry was performed as previously described.^23^

### Xenograft tumour formation

Experiments with animals were performed in accordance to the guidelines for experimental animal managements established by Kagawa University and guidelines for the welfare and use of animals in cancer research.^33^ Five-week-old male BALB/c nude mice (nu+/nu+) were purchased from CREA (Shizuoka, Japan). Mice were anesthetised with sevoflurane. DLD-1 cells (2 × 10^6^ cells) transfected with either (P)RR shRNA or scrambled shRNA were suspended in 200 μL PBS and subcutaneously injected into the upper right flanks. Sizes of xenograft tumours were measured by an electric calliper and tumour volumes were calculated based on the formula: volume (mm^3^) = length (mm) × width (mm) × width (mm)/2. Mice were sacrificed by overdose of pentobarbital (250 mg/kg, intraperitoneal injection) and the tumours were then taken out and used for IHC as previously described.^26^

### Statistical analysis

For numerical data, two groups were compared by unpaired *t* test, while three groups were compared using one-way ANOVA followed by Tukey’s test. Constituent ratios were compared by Chi-square test. Survival curves were generated by Kaplan–Meier method and analysed by Log-rank (Mantel-Cox) test. A *P*-value < 0.05 was considered statistically significant. All statistical analyses were performed using Prism software (GraphPad Software, La Jolla, CA, USA, version 5).

## Results

### (P)RR is highly expressed in human CRC tissues, especially those with excessive activation of Wnt/β-catenin pathway

**Fig. 1:**
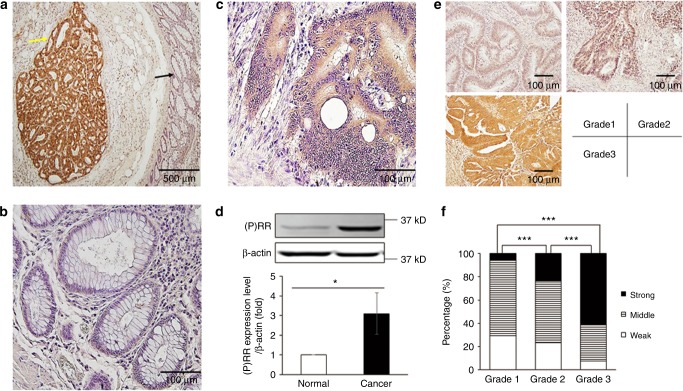
(P)RR is highly expressed in human CRC tissues. **a** Representative image of (P)RR staining by IHC in colorectal tissues, which contains both cancer and normal tissues. Yellow arrow: cancer tissue with messy and irregular gland structure. Black arrow: normal tissue with regular gland structure. **b**, **c** Representative IHC images of (P)RR staining in normal (**b**) and cancer (**c**) tissues with higher magnification. **d** Western blotting analysis of (P)RR expression levels in cancer and matched normal tissues (*n* = 5). Data are presented as mean ± SEM. **e** Representative IHC images of (P)RR staining in CRC tissues with different differentiated grades. **f** Comparison of percentages of patients showing strong, middle or weak (P)RR expression in groups with different CRC grades (*n* = 17, 30 and 13 in Grade 1, 2 and 3). *P *<* 0.05, ***P *<* 0.001

**Fig. 2:**
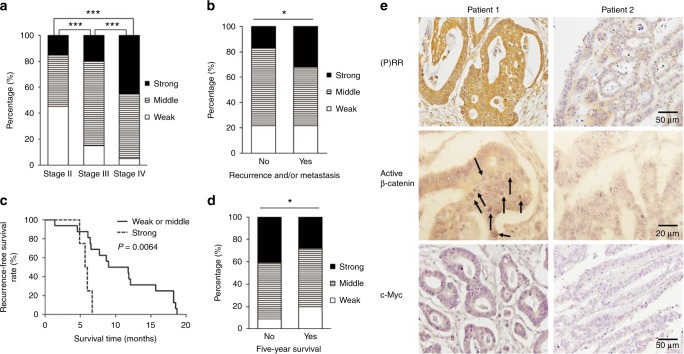
(P)RR level in CRC is related to clinical characteristics and the activation of Wnt/β-catenin signalling. **a** Comparison of percentages of patients showing strong, middle or weak (P)RR expression in different stages (*n* = 20 in each stage). **b** Comparison of percentages of patients showing strong, middle or weak (P)RR expression in groups with and without recurrence and/or metastasis to other organ(s) (*n* = 23 without recurrence and metastasis; *n* = 37 with recurrence and/or metastasis). **c** Relevancy between (P)RR expression level and recurrence-free survival time of 20 patients in Stage II (*n* = 10) and III (*n* = 10) who had recurrence after radical operation (*n* = 16 with weak or middle (P)RR expression; *n* = 4 with strong (P)RR expression). **d** Comparison of percentages of patients showing strong, middle or weak (P)RR expression in groups with and without a 5-year survival (5-year follow-up after diagnosis of CRC was accomplished on 47 patients; 25 patients had survived over 5 years; 22 patients died within 5 years). **e** Representative IHC images of active β-catenin and c-Myc staining in CRC tissues with different (P)RR levels. Black arrow: staining of active β-catenin translocated into nuclei. *P *<* 0.05, ***P *<* 0.001

We first performed IHC staining of (P)RR with human colorectal tissue sections that included both cancer and normal regions. Cancer tissue showed aberrantly high (P)RR expression, while normal epithelium showed very weak (P)RR staining (Fig. 1a–c). As the negative control, almost no staining by the rabbit serum, from which the (P)RR antibody was isolated, was seen in both normal and cancer regions (Supplementary Figure S1), confirming that (P)RR staining with its antibody was highly specific. Western blotting analysis of (P)RR expression levels in CRC and the matched normal tissues from five patients showed that (P)RR protein level in cancer tissues was on average 3.1-fold of that in normal tissues (*P* *<* 0.05) (Fig. 1d). We also assessed (P)RR expression in cancers of different grades. CRCs were classified using the WHO criteria into Grade 1, 2 and 3, which indicates well-, moderately-, and poorly-differentiated cancers, respectively. We found that (P)RR expression was generally the lowest in Grade 1 cancers, at the middle level in Grade 2 cancers, and the highest in Grade 3 cancers (Fig. 1e). Moreover, in comparison with cancers of lower grade, cancers of higher grades showed greater proportions of lesions with strong (P)RR expression and smaller proportions with weak (P)RR expression (Fig. 1f). In grade 1 cancers, 29% showed weak (P)RR expression compared with 23 and 8% in Grade 2 and 3 cancers. In contrast, 6% of Grade 1 cancers showed strong (P)RR expression compared with 23 and 62% in Grade 2 and 3 cancers. Taken together, these data reveal that aberrantly strong expression of (P)RR is associated to poor differentiation of CRC.

(P)RR expression was also assessed in CRCs of patients with different clinical characteristics. Compared with CRCs in an earlier stage, those in more advanced stages showed larger proportions of cancers with strong and middle (P)RR expression and smaller proportions with weak (P)RR expression (Fig. 2a). In Stage II, 45% of the cancers showed weak (P)RR expression compared with 15 and 5% in Stage III and IV. In contrast, 15% of cancers in Stage II showed strong (P)RR expression compared with 20 and 45% in Stage III and Stage IV. In Stage III, the most cancers (65%) showed middle (P)RR expression compared with 40 and 50% in Stage II and IV. Patients who had recurrence within 5 years after radical operation and/or metastasis to other organ(s) showed a higher proportion of lesions with strong (P)RR expression than those without recurrence and metastasis (32% vs. 17%) (Fig. 2b). Among the 20 patients in Stage II and III who had cancer recurrence after radical surgical resection, the percentages of patients with different recurrence-free survival time (duration from surgery to diagnosis of recurrence) were calculated. Compared with patients showing weak or middle (P)RR expression, those with strong (P)RR expression had a shorter recurrence-free survival time (median value: 10.4 vs. 5.85 months; *P* *=* 0.0064, *χ*^2^ = 7.438, HR = 0.06804, 95% CI = 0.009861 to 0.4695) (Fig. 2c). Patients who reached a 5-year survival showed a smaller proportion of lesions with strong (P)RR expression and a higher proportion of lesions with weak (P)RR expression, in comparison to those died within 5 years after diagnosis of CRC (28% vs. 41% of strong expression; 20% vs. 9% of weak expression) (Fig. 2d). Overall, strong expression of (P)RR in CRC may predict the high risk of rapid progression and poor prognosis.

Given that CRC is closely associated with the activation of Wnt/β-catenin signalling, levels of active β-catenin (non-phosphorylated form) and the Wnt target protein c-Myc were measured in CRC tissues with different (P)RR levels. In comparison with tissues that showed lower (P)RR levels, tissues with higher (P)RR levels also showed elevated levels of active β-catenin and c-Myc, especially increased β-catenin translocation into nuclei (Fig. 2e).

### (P)RR silencing attenuates the activation of Wnt/β-catenin signalling in CRC cells

**Fig. 3:**
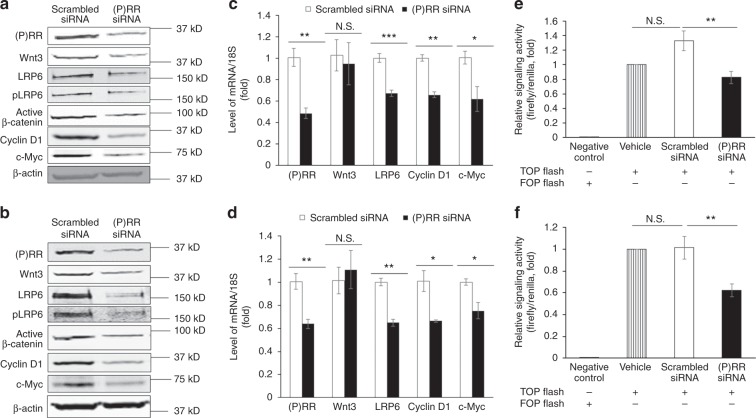
(P)RR silencing attenuates the activation of Wnt/β-catenin signalling in CRC cells. **a**, **b** Representative western blotting images showing protein levels of components of the Wnt/β-catenin pathway in DLD-1 cells with mutated APC (**a**) and HCT116 cells with mutated β-catenin (**b**) after siRNA transfection. **c**, **d** Relative mRNA levels of components of the Wnt/β-catenin pathway in DLD-1 (**c**) and HCT116 (**d**) cells after siRNA transfection. Values were calculated from signal intensities obtained by real-time PCR (*n* = 3~6). Data are expressed as relative ratios to the scrambled siRNA group. **e**, **f** Wnt/β-catenin signalling activity of DLD-1 (**e**) and HCT116 (**f**) cells transfected with β-catenin-responsive TOP flash reporter and siRNA (*n* = 4). Data are expressed as relative ratios to vehicle (only transfected with the TOP flash reporter without siRNA) group. Cells transfected with the FOP flash reporter, of which the TCF/LEF binding sites were mutated, served as negative control. Numerical data are presented as mean ± SEM. N.S.: not significant, *P *<* 0.05, **P *<* 0.01, ***P *<* 0.001

We next investigated whether (P)RR plays a role in the Wnt/β-catenin pathway in CRC cells. We checked the genetic characterisations of over 30 commercially available human CRC cell lines in the Cancer Cell Line Encyclopaedia and Cosmic databases and found that all these cell lines contain mutation in the Wnt/β-catenin pathway. Two representative human CRC cell lines,^19^ DLD-1 with APC truncated mutation and HCT116 with β-catenin activating mutation (loss of phosphorylation site S45), were widely used in experiments. A recent study has indicated that silencing of Wnt3 attenuated the activity of Wnt/β-catenin signalling in both DLD-1 and HCT116 cells.^19^ Therefore, we first examined the effect of (P)RR on the endogenous Wnt3 protein level of CRC cells. We found that (P)RR silencing remarkably decreased protein levels of not only Wnt3, but also total LRP6 and phosphorylated-LRP6 (pLRP6), the major components of the Wnt receptor complex, in both DLD-1 (Fig. 3a) and HCT116 (Fig. 3b) cells. (P)RR silencing also obviously reduced protein levels of active β-catenin, as well as Wnt target proteins Cyclin D1 and c-Myc (Fig. 3a, b). We next asked whether (P)RR could affect the mRNA levels of the pathway components. Interestingly, (P)RR silencing showed no impact on Wnt3 mRNA level compared with the scrambled siRNA group, but significantly decreased mRNA levels LRP6, Cyclin D1 and c-Myc in both DLD-1 (Fig. 3c) and HCT116 (Fig. 3d) cells. In summary, these data suggest that (P)RR is involved in the up-regulation of Wnt3 at the post-transcriptional level and LRP6 at the transcriptional level, which may further contribute to the activation of Wnt/β-catenin signalling and its target oncogene expression in CRC cells.

To directly measure Wnt/β-catenin signalling activity, luciferase assay was performed. Data showed that (P)RR silencing obviously reduced the signalling activity in both DLD-1 (Fig. 3e) and HCT116 (Fig. 3f) cells, which were transfected with the TOP flash vector. As negative controls, CRC cells transfected with the FOP flash vector, of which the TCF/LEF binding sites were mutated, showed nearly no activity (Fig. 3e, f).

We then explored the molecular mechanism through which (P)RR regulates Wnt3 protein level. Firstly, to further confirm the effects of (P)RR silencing on Wnt3 expression, we immunoprecipitated Wnt3 protein from the whole lysates of DLD-1 cells, which were transfected with either scrambled siRNA or (P)RR siRNA, followed by immunoblotting (IB) with Wnt3 antibody. Data confirmed that the amount of immunoprecipitated Wnt3 protein was much less in the (P)RR silencing group than control group (Supplementary Figure S2A). To examine the potential binding interaction between (P)RR and Wnt3, we immunoprecipitated Wnt3 protein from the whole lysates of DLD-1 cells, followed by IB with (P)RR antibody. However, (P)RR was not detected in the Wnt3 immunocomplex (Supplementary Figure S2B), indicating no direct binding between (P)RR and Wnt3. Thus far, the detailed molecular mechanism through which (P)RR regulates Wnt3 protein level remains unknown, and further research to elucidate this regulatory mechanism is required in the future.

### (P)RR silencing inhibits proliferation and induces apoptosis of CRC cells in vitro

**Fig. 4:**
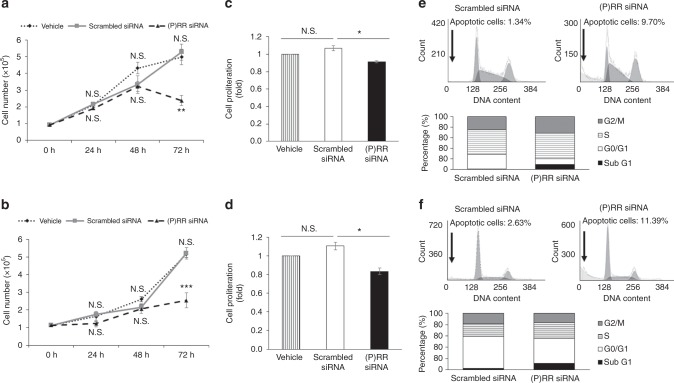
(P)RR silencing inhibits proliferation and induces apoptosis of CRC cells in vitro. **a**, **b** Cell number counting of DLD-1 (**a**) and HCT116 (**b**) cells during 72 h after siRNA transfection (*n* = 4). N.S.: not significant, **P *<* 0.01, ***P *<* 0.001 vs. scrambled siRNA group. **c**, **d** Proliferative activity of DLD-1 (**c**) and HCT116 (**d**) cells measured by WST-1 assay at 72 h after siRNA transfection (*n* = 6). Data are expressed as relative ratios to vehicle (without siRNA transfection) group. N.S.: no significant difference, *P *<* 0.05. **e**, **f** Flow cytometry detection of apoptosis and cell cycle distribution of DLD-1 (**e**) and HCT116 (**f**) cells at 72 h after siRNA transfection. Numerical data are presented as mean ± SEM

Cell numbers were counted, and colony formation was observed during 72 h after siRNA transfection into CRC cells. Equal number of cells were initially seeded into each group before siRNA transfection. In both DLD-1 and HCT116 cells, compared with scrambled siRNA group, (P)RR silencing significantly inhibited the cell number increase (Fig. 4a, b) and colony formation (Supplementary Figure S3). Next, we further investigated the specific mechanisms in CRC cell number regulation by (P)RR. Cell proliferative activity was measured by WST-1 assay. Compared with the scrambled siRNA group, (P)RR silencing markedly repressed the proliferative activity of both DLD-1 (Fig. 4c) and HCT116 (Fig. 4d) cells at 72 h after siRNA transfection. Additionally, in both DLD-1 (Fig. 4e) and HCT116 (Fig. 4f) cells, an obvious sub-G1 apoptotic peak was detected at 72 h after (P)RR siRNA transfection by flow cytometry, and the percentage of apoptotic cells was much higher in the (P)RR silencing group than scrambled siRNA group. Together this suggests that (P)RR contributes to cell proliferation and ameliorates apoptosis of CRC cells in vitro.

### (P)RR silencing attenuates growth and Wnt/β-catenin signalling of CRC xenografts in vivo

**Fig. 5:**
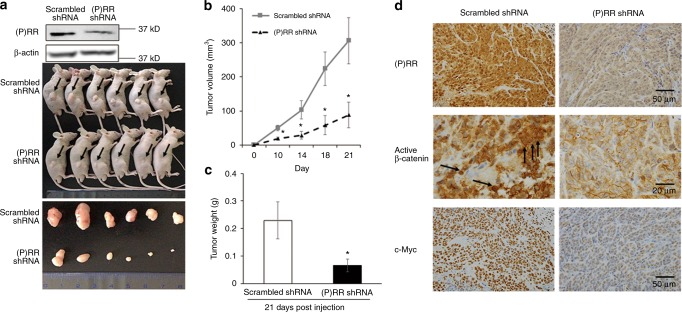
(P)RR silencing attenuates growth and Wnt/β-catenin signalling of CRC xenografts in vivo. **a** Tumour formation of scrambled shRNA- and (P)RR shRNA-transfected DLD-1 cells at day 21 after subcutaneous transplantation into nude mice (*n* = 6). Successful (P)RR silencing was confirmed by western blotting before cell transplantation. **b** Growth patterns of tumour volume during 21 days. **c** Tumour weights at day 21. **d** Representative IHC images of (P)RR, active β-catenin and c-Myc staining in xenograft tumour tissues. Black arrow: staining of active β-catenin translocated into nuclei. Numerical data are presented as mean ± SEM. *P *<* 0.05 vs. scrambled shRNA group

We also examined the effects of (P)RR silencing on the growth of xenograft tumours in vivo. DLD-1 cells (2 × 10^6^ cells) transfected with either scrambled shRNA or (P)RR shRNA were subcutaneously transplanted into nude mice, and tumour sizes were measured weekly. Successful (P)RR silencing was confirmed by western blotting before cell transplantation (Fig. 5a). All mice were sacrificed at day 21 after cell transplantation and then tumours were taken out (Fig. 5a). Compared with scrambled shRNA group, the average tumour volume in the (P)RR silencing group was obviously smaller from day 10 to day 21 (Fig. 5b). Moreover, the average tumour weight in the (P)RR silencing group at day 21 was much less than scrambled shRNA group (Fig. 5c). In addition, levels of (P)RR, active β-catenin and c-Myc were assessed in xenograft tumour tissues. Compared with tissues from scrambled shRNA group, tissues from the (P)RR shRNA group showed lower levels of (P)RR, active β-catenin and c-Myc, especially decreased β-catenin translocation into nuclei. Representative images are shown in Fig. 5d. These data indicate that (P)RR play a role in the progression and Wnt/β-catenin signalling in CRC in vivo.

### Induction of (P)RR overexpression enhances the Wnt/β-catenin signalling and proliferation of normal colon epithelial cells

**Fig. 6:**
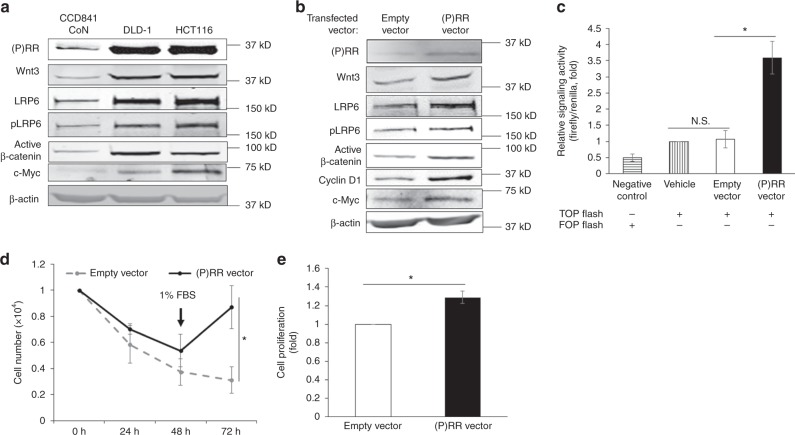
Induction of (P)RR overexpression enhances the Wnt/β-catenin signalling and proliferation of normal colon epithelial cells. **a** Representative western blotting images showing expression levels of components of the Wnt/β-catenin pathway in CCD841 CoN cells, a normal colon epithelial cell line, as well as DLD-1 and HCT116 cells. **b** Representative western blotting images showing expression levels of components of the Wnt/β-catenin pathway in CCD841 CoN cells, transfected with either plasmid vector containing (P)RR encoding gene *ATP6ap2* ((P)RR vector) or empty vector. **c** Wnt/β-catenin signalling activity of CCD841 CoN cells transfected with β-catenin-responsive TOP flash reporter as well as (P)RR vector or empty vector (*n* = 4). Data are expressed as relative ratios to vehicle (only transfected with the TOP flash reporter) group. Cells transfected with the FOP flash reporter, of which the TCF/LEF binding sites were mutated, served as negative control. **d** Cell number counting of CCD841 CoN cells during 72 h after vector transfection (*n* = 3). **e** Proliferative activity of CCD841 CoN cells measured by WST-1 assay at 72 h after vector transfection. Numerical data are presented as mean ± SEM. N.S.: not significant, *P < 0.05

Given that excessive activation of Wnt/β-catenin signalling and aberrant cell proliferation are regarded as the initial events of cancerisation from normal. We asked whether induction of (P)RR overexpression in a normal colon epithelial cell line, CCD841 CoN, could promote the Wnt/β-catenin signalling and cell proliferation. First, we compared basal expression levels of (P)RR and other components of the Wnt/β-catenin pathway in CCD841 CoN and CRC cells. Compared with CCD841 CoN cells, the expression levels of (P)RR, Wnt3, LRP6, pLRP6, active β-catenin and c-Myc were significantly higher in DLD-1 and HCT116 cells (Fig. 6a). We next transfected CCD841 CoN cells with either plasmid vector containing (P)RR encoding gene *ATP6AP2* ((P)RR vector) or empty vector. Successful induction of (P)RR overexpression was confirmed in the (P)RR vector-transfected cells in comparison to empty vector-transfected cells (Fig. 6b). Furthermore, (P)RR overexpression markedly enhanced expression levels of Wnt3, LRP6, pLRP6, active β-catenin, as well as Wnt target proteins Cyclin D1 and c-Myc (Fig. 6b). We also measured Wnt/β-catenin signalling activity. We found that the basal activity level in vehicle CCD841 CoN cells was very low, and empty vector-transfected cells showed a similar activity level (Fig. 6c). However, (P)RR overexpression remarkably elevated the signalling activity (Fig. 6c).

Cell numbers were also monitored during 72 h after plasmid vector transfection into CCD841 CoN cells. Equal number of cells were initially seeded into each group before vector transfection. Given that serum-induced cell proliferation might obscure the effect of (P)RR overexpression, cells were maintained in serum-free condition in the first 2 days after vector transfection. Unlike cancer cells, the transfected normal epithelial cells were extremely vulnerable to the serum-free condition. Therefore, a continuous cell number decrease was observed in the first 2 days in both empty vector and (P)RR vector groups (Fig. 6d). To improve the cell condition, we added 1% foetal bovine serum into culture medium at 48 h after transfection. Cell number was seen to increase in the (P)RR vector group at 72 h while it continuously decreased in empty vector group, and the difference was significant (Fig. 6d). WST-1 assay confirmed the higher proliferative activity of cells in the (P)RR vector group than empty vector group at 72 h (Fig. 6e). Together these data indicate that induction of (P)RR overexpression in normal colon epithelial cells promotes activation of Wnt/β-catenin signalling and cell proliferation.

## Discussion

The present study has demonstrated, for the first time, that (P)RR promotes human CRC through the Wnt/β-catenin signalling pathway despite the presence of constitutive pathway-activating mutations. Our results reveal four novel findings. First, aberrant (P)RR expression in human CRC is correlated to patients’ clinicopathologic characteristics and prognosis. Second, (P)RR silencing attenuates the activation of Wnt/β-catenin signalling in human CRC cells, despite the presence of constitutive APC loss-of-function or β-catenin activating mutation. Third, (P)RR silencing inhibits proliferation and induces apoptosis of human CRC cells. Finally, induction of (P)RR overexpression in normal human colon epithelial cells enhances the Wnt/β-catenin signalling and promotes cell proliferation. These data suggest that (P)RR is a potential biomarker for diagnosis and progression prediction, as well as a therapeutic target of CRC.

The TNM classification is widely used for staging patients and selecting the specific treatments; however, it fails to accurately predict the progressing speed and prognosis of patients who have received curative surgery for localised CRC.^34^ In fact, 10–20% of patients with stage II CRC and 30–40% of patients with stage III CRC develop recurrence after surgical excision.^34^ Recently, Arundhathi et al.^35^ have reported that (P)RR expression level is positively associated with the TNM stage of pancreatic ductal adenocarcinoma. In the present study, we have generally observed stronger (P)RR expression in tissues of poorly-differentiated, advanced and/or early-progressing CRCs. Moreover, CRC patients with strong (P)RR expression had a relatively shorter recurrence-free survival time. These findings suggest that (P)RR is a potential predictive indicator of the severity and prognosis of CRC.

Constitutive activating mutations in the Wnt/β-catenin pathway play an essential role in the pathogenesis of CRC.^19^ (P)RR is proved crucial for Wnt/β-catenin-dependent tumourigenesis of pancreatic ductal adenocarcinoma^23^ and glioma.^24^ In the present study, we found that (P)RR level in CRC tissues showed a positive correlation with levels of active β-catenin and c-Myc, especially β-catenin translocation into nuclei. Furthermore, in CRC cell lines with APC loss-of-function mutation or β-catenin activating mutation, (P)RR silencing decreased Wnt3 protein level, as well as protein and mRNA levels of total LRP6, pLRP6, active β-catenin, Cyclin D1 and c-Myc. (P)RR silencing also attenuated CRC cell proliferation and induced apoptosis in vitro, and inhibited tumour growth in vivo. On the other hand, induction of (P)RR overexpression aberrantly activated the Wnt/β-catenin signalling and proliferation of normal colon epithelial cells. In agreement with our findings, Voloshanenko et al.^19^ demonstrated that Wnt3 expression is frequently higher in colon adenomas and carcinomas than in normal colon epithelium; Silencing of Wnt3 significantly decreased the activity of Wnt/β-catenin pathway in CRC cells with APC or β-catenin mutation and their proliferation. Additionally, downregulation of (P)RR or Wnt3 induces cell apoptosis of glioma, pancreatic and gastric cancers by attenuating Wnt/β-catenin activity.^23,24,36^ In regard to the molecular mechanism responsible for the (P)RR-mediated Wnt3 regulation, we found that Wnt3 mRNA level was not affected by (P)RR silencing, suggesting that (P)RR regulates Wnt3 at the post-transcriptional level. We could not detect any binding between Wnt3 and (P)RR. Thus, the detailed molecular mechanism underlying the regulation of Wnt3 by (P)RR remains unclear and further studies are required in the future.

Our data also showed that protein level of LRP6, a crucial element of the Wnt receptor complex, was remarkably higher in CRC cells than normal colon epithelial cells. This is consistent with the previous finding showing higher LRP6 expression in colon tumour tissues than normal colon tissues.^37^ Liu et al.^37,38^ reported that suppression of LRP6 expression inhibits Wnt/β-catenin signalling and tumour growth even when Wnt ligands are augmented. In the present study, we further found that (P)RR repression is an effective way to down-regulate LPR6 at both protein and mRNA levels. Collectively, these data support our hypothesis that (P)RR augments both Wnt3 and LRP6 protein expression, which will synergistically activate the Wnt/β-catenin pathway (Supplementary Figure S4).

Although multiple studies have demonstrated the effect of (P)RR on promoting Wnt/β-catenin signalling in various cell lines, opposite results were observed in human embryonic kidney HEK293 cells and human hepatoma HepG2 cells.^39^ We speculate that there may be other unknown components that interact with (P)RR and influence its impact on the pathway signalling, thus the heterogeneity of their expression in cells of different origins may cause the difference. Therefore, further studies should be needed in the future.

In conclusion, the present study demonstrates that aberrant (P)RR expression promotes the carcinogenesis of CRC through the Wnt/β-catenin signalling pathway despite the presence of constitutive pathway component mutations. Therefore, (P)RR is a potential biomarker for diagnosis and progression prediction, as well as a promising therapeutic target of CRC.

## Electronic supplementary material

Supplementary Table

Supplementary Figures

## Availability of data and materials

All data generated or analysed during this study are included in this article and its supplementary information files.
