# Supplementary material for: (Pro)renin receptor promotes colorectal cancer through the Wnt/beta-catenin signalling pathway despite constitutive pathway component mutations
Source: Br J Cancer. 2018 Dec 17;120(2):229–37. doi: 10.1038/s41416-018-0350-0 (PMC6342928; doi:10.1038/s41416-018-0350-0)
Supplement: Supplementary file 1 — Supplementary Table [file 41416_2018_350_MOESM1_ESM.doc]

| **Supplementary Table S1. Clinicopathologic characteristics of the 60 enrolled patients with CRC** | | | | | | |
| --- | --- | --- | --- | --- | --- | --- |
| **Patient**  **number** | **Age**  **(years)** | **Gender** | **Stage**  **(UICC)** | **Grade**  **(WHO)** | **Histological**  **type** | **Maximum diameter of tumor**  **(mm)** |
| 1 | 66 | F | Ⅱ | 2 | Tub | 24 |
| 2 | 60 | M | Ⅱ | 3 | Tub | 50 |
| 3 | 57 | F | Ⅱ | 2 | Tub | 30 |
| 4 | 66 | F | Ⅱ | 3 | Tub | 90 |
| 5 | 60 | F | Ⅱ | 2 | Tub | 80 |
| 6 | 73 | M | Ⅱ | 2 | Muc | 50 |
| 7 | 75 | M | Ⅱ | 3 | Tub | 50 |
| 8 | 82 | M | Ⅱ | 2 | Tub | 15 |
| 9 | 60 | F | Ⅱ | 2 | Tub | 25 |
| 10 | 79 | M | Ⅱ | 2 | Tub | 40 |
| 11 | 78 | M | Ⅲ | 2 | Tub | 40 |
| 12 | 68 | F | Ⅲ | 2 | Tub | 100 |
| 13 | 65 | M | Ⅲ | 2 | Tub | 25 |
| 14 | 73 | F | Ⅲ | 2 | Tub | 15 |
| 15 | 49 | F | Ⅲ | 2 | Tub | 50 |
| 16 | 75 | M | Ⅲ | 3 | Tub | 30 |
| 17 | 69 | F | Ⅲ | 2 | Tub | 50 |
| 18 | 82 | F | Ⅲ | 1 | Tub | 65 |
| 19 | 67 | F | Ⅲ | 3 | Tub | 55 |
| 20 | 75 | M | Ⅲ | 3 | Tub | 35 |
| 21 | 70 | M | Ⅳ | 3 | Tub | 40 |
| 22 | 71 | M | Ⅳ | 3 | Muc | 60 |
| 23 | 66 | F | Ⅳ | 3 | Tub | 50 |
| 24 | 57 | F | Ⅳ | 3 | Tub | 50 |
| 25 | 62 | M | Ⅳ | 2 | Tub | 60 |
| 26 | 66 | F | Ⅳ | 3 | Tub | 65 |
| 27 | 52 | F | Ⅳ | 1 | Muc | 35 |
| 28 | 58 | M | Ⅳ | 2 | Tub | 50 |
| 29 | 79 | M | Ⅳ | 3 | Tub | 75 |
| 30 | 68 | M | Ⅳ | 3 | Tub | 45 |
| 31 | 74 | F | Ⅱ |  | Tub | 26 |
| 32 | 50 | M | Ⅱ |  | Tub | 75 |
| 33 | 72 | F | Ⅱ |  | Tub + Muc | 70 |
| 34 | 57 | F | Ⅱ |  | Tub | 70 |
| 35 | 71 | F | Ⅱ |  | Tub + Muc | 55 |
| 36 | 70 | M | Ⅱ |  | Tub | 70 |
| 37 | 75 | M | Ⅱ |  | Tub | 86 |
| 38 | 65 | F | Ⅱ |  | Tub | 100 |
| 39 | 87 | F | Ⅱ |  | Tub + Muc | 90 |
| 40 | 67 | M | Ⅱ |  | Tub | 120 |
| 41 | 68 | M | Ⅲ |  | Tub | 60 |
| 42 | 69 | M | Ⅲ |  | Tub | 18 |
| 43 | 83 | F | Ⅲ |  | Tub | 45 |
| 44 | 60 | M | Ⅲ |  | Tub | 70 |
| 45 | 75 | M | Ⅲ |  | Tub | 60 |
| 46 | 65 | M | Ⅲ |  | Tub | 45 |
| 47 | 58 | M | Ⅲ |  | Tub | 40 |
| 48 | 75 | M | Ⅲ |  | Tub | 20 |
| 49 | 74 | M | Ⅲ |  | Tub | 80 |
| 50 | 63 | F | Ⅲ |  | Tub | 60 |
| 51 | 59 | M | Ⅳ |  | Tub | 70 |
| 52 | 60 | M | Ⅳ |  | Tub | 65 |
| 53 | 77 | F | Ⅳ |  | Tub | 100 |
| 54 | 69 | M | Ⅳ |  | Tub | 50 |
| 55 | 67 | F | Ⅳ |  | Tub | 45 |
| 56 | 65 | M | Ⅳ |  | Tub | 30 |
| 57 | 44 | F | Ⅳ |  | Muc | 130 |
| 58 | 53 | F | Ⅳ |  | Tub | 30 |
| 59 | 56 | M | Ⅳ |  | Tub + Muc | 45 |
| 60 | 70 | M | Ⅳ |  | Tub | 60 |

Abbreviations: M, Male; F, Female; UICC, Union for International Cancer Control; WHO, World Health Organization; Tub, Tubular adenocarcinoma; Muc, mucinous adenocarcinoma
