# Supplementary material for: (Pro)renin receptor promotes colorectal cancer through the Wnt/beta-catenin signalling pathway despite constitutive pathway component mutations
Source: Br J Cancer. 2018 Dec 17;120(2):229–37. doi: 10.1038/s41416-018-0350-0 (PMC6342928; doi:10.1038/s41416-018-0350-0)
Supplement: Supplementary file 2 — Supplementary Figures [file 41416_2018_350_MOESM2_ESM.pdf]

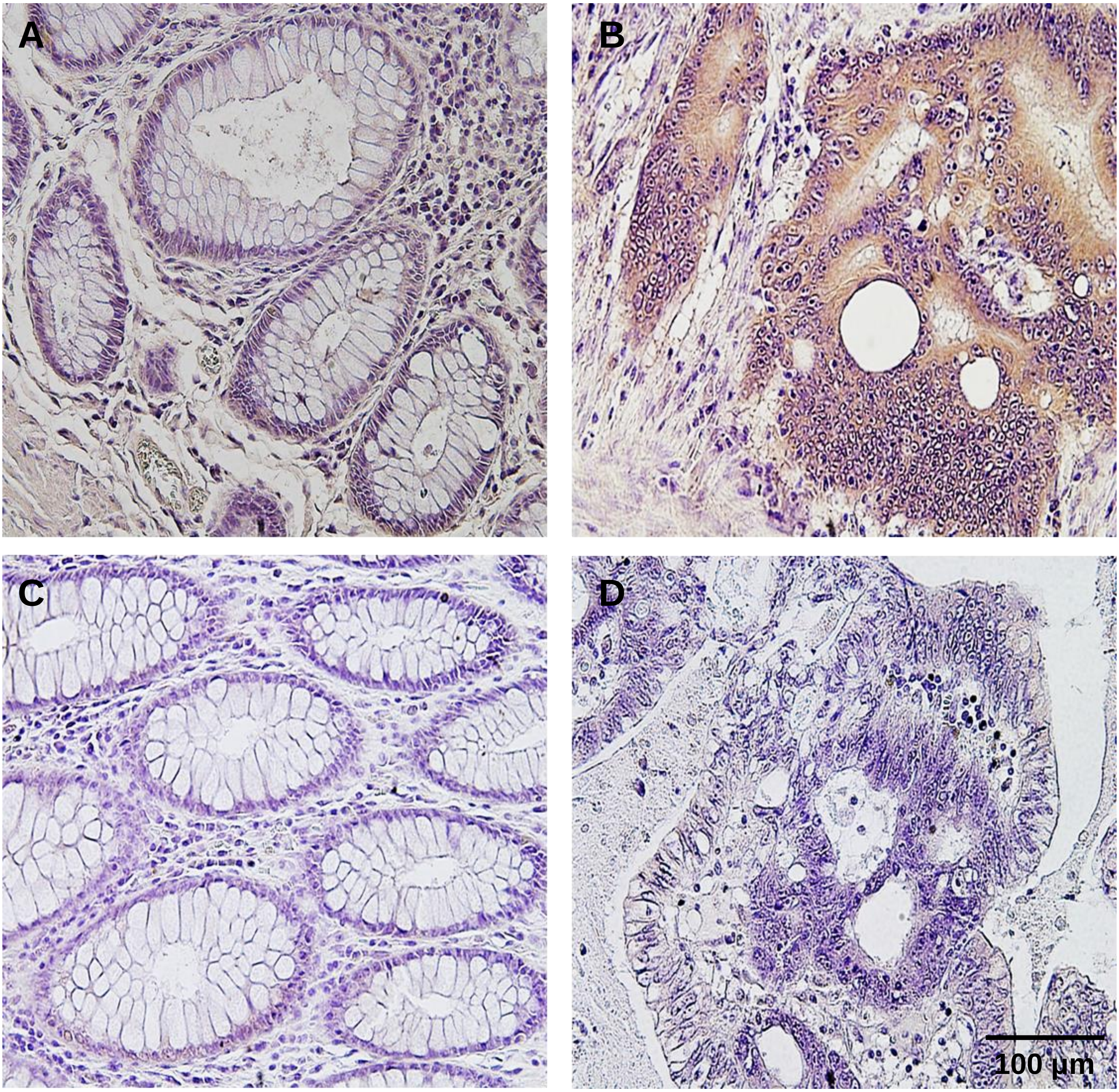

Supplementary Figure S1. Confirmation of the specificity of immunohistochemistry staining with (P)RR antibody in human colorectal tissues. (**A** and **B**) Staining with (P)RR antibody in normal (**A**) and cancer (**B**) regions. (**C** and **D**) As the negative control, almost no obvious staining with rabbit serum, from which (P)RR antibody was isolated, was seen in normal (**C**) and cancer (**D**) regions.

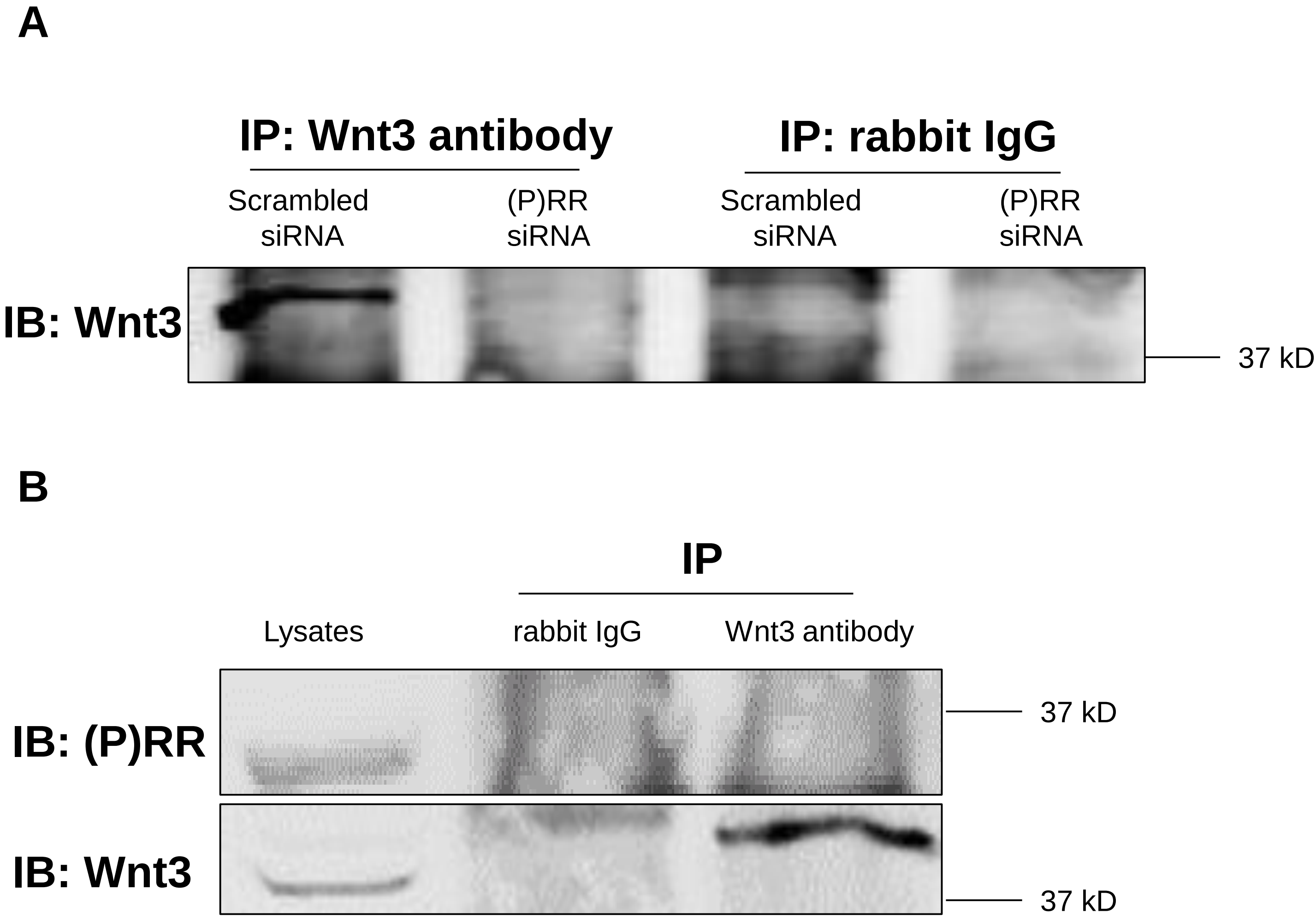

Supplementary Figure S2. (P)RR affects Wnt3 protein level without direct binding interaction. (**A**) Decreased Wnt3 protein level in DLD-1 cells after (P)RR silencing was confirmed through immunoprecipitation. (**B**) (P)RR did not directly bind with Wnt3. (P)RR was undetectable in the Wnt3 immunocomplex. Whole cell lysate served as positive control. Rabbit IgG served as negative control. IP: immunoprecipitation; IB: immunoblotting.

A

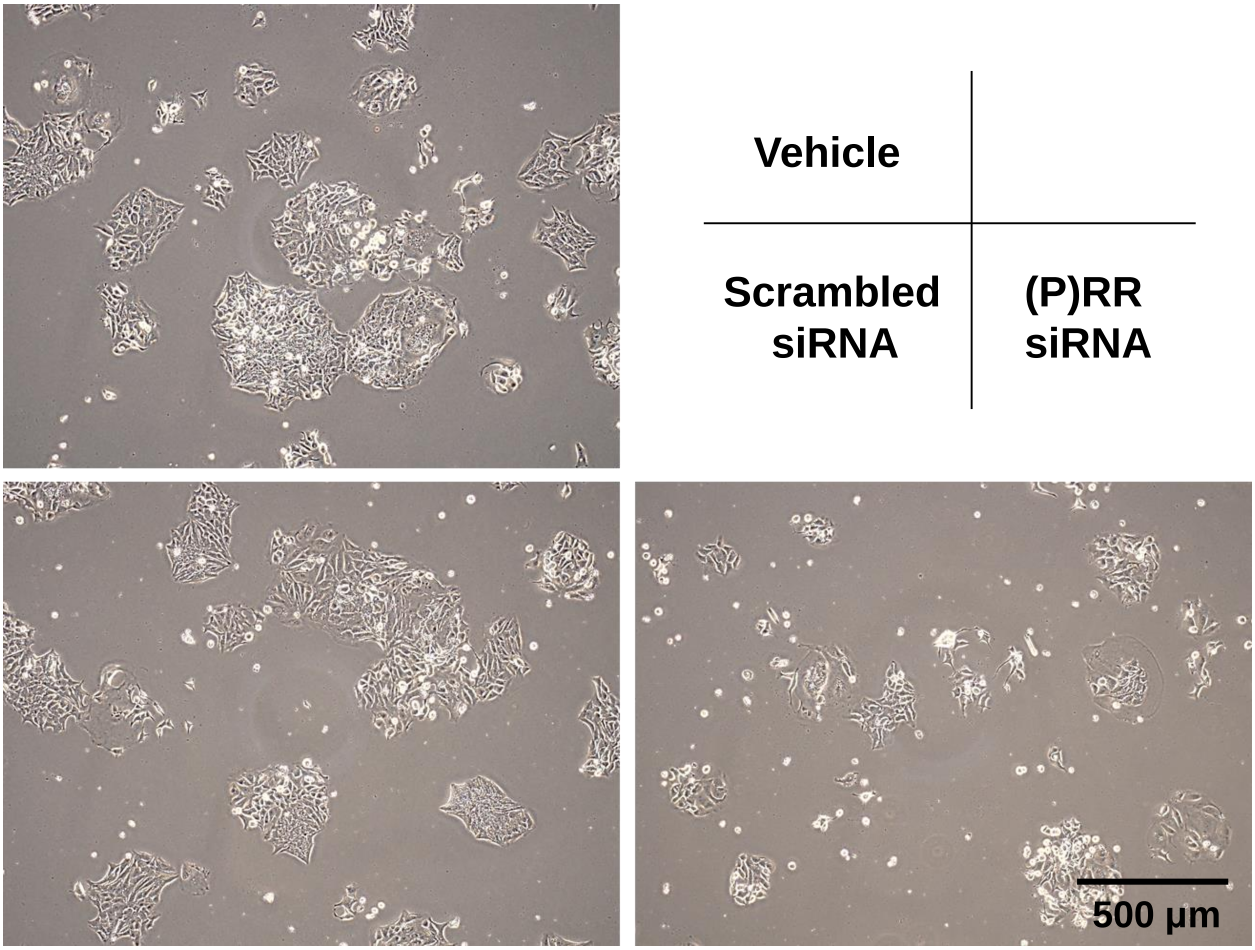
Vehicle
Scrambled siRNA
(P)RR siRNA
500 μm

B

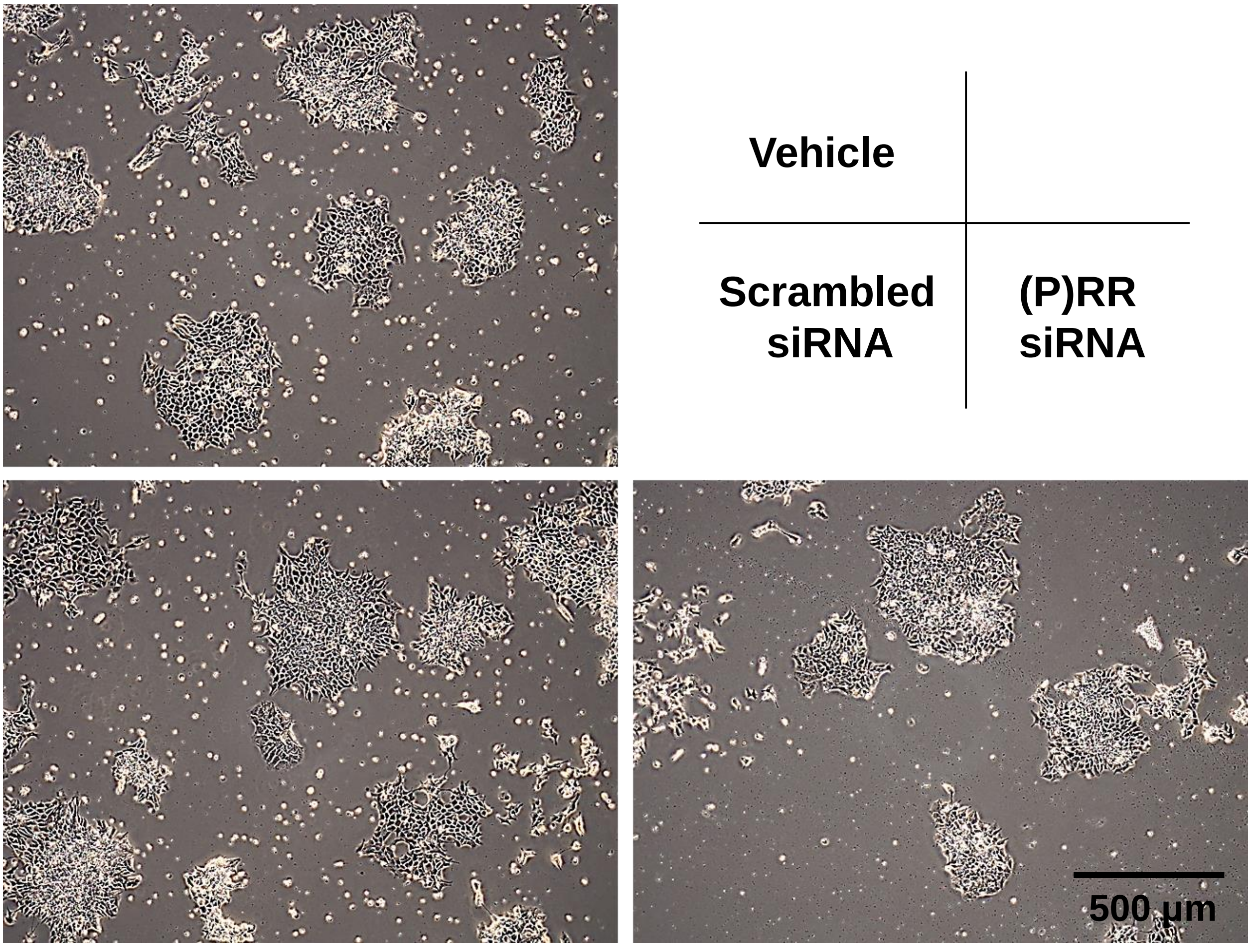
Vehicle
Scrambled siRNA
(P)RR siRNA
500 μm

Supplementary Figure S3. (P)RR silencing attenuates colony formation of colorectal cancer cells. (**A** and **B**) Equal number of DLD-1 (**A**) and HCT116 (**B**) cells were initially seeded into each group. Then, cells were respectively transfected with scrambled siRNA or (P)RR siRNA, or without any treatment (vehicle), followed by the observation of colony formation after 72 h. In comparison with vehicle and scrambled siRNA groups, colony formation of both cell lines was obviously attenuated by (P)RR silencing.

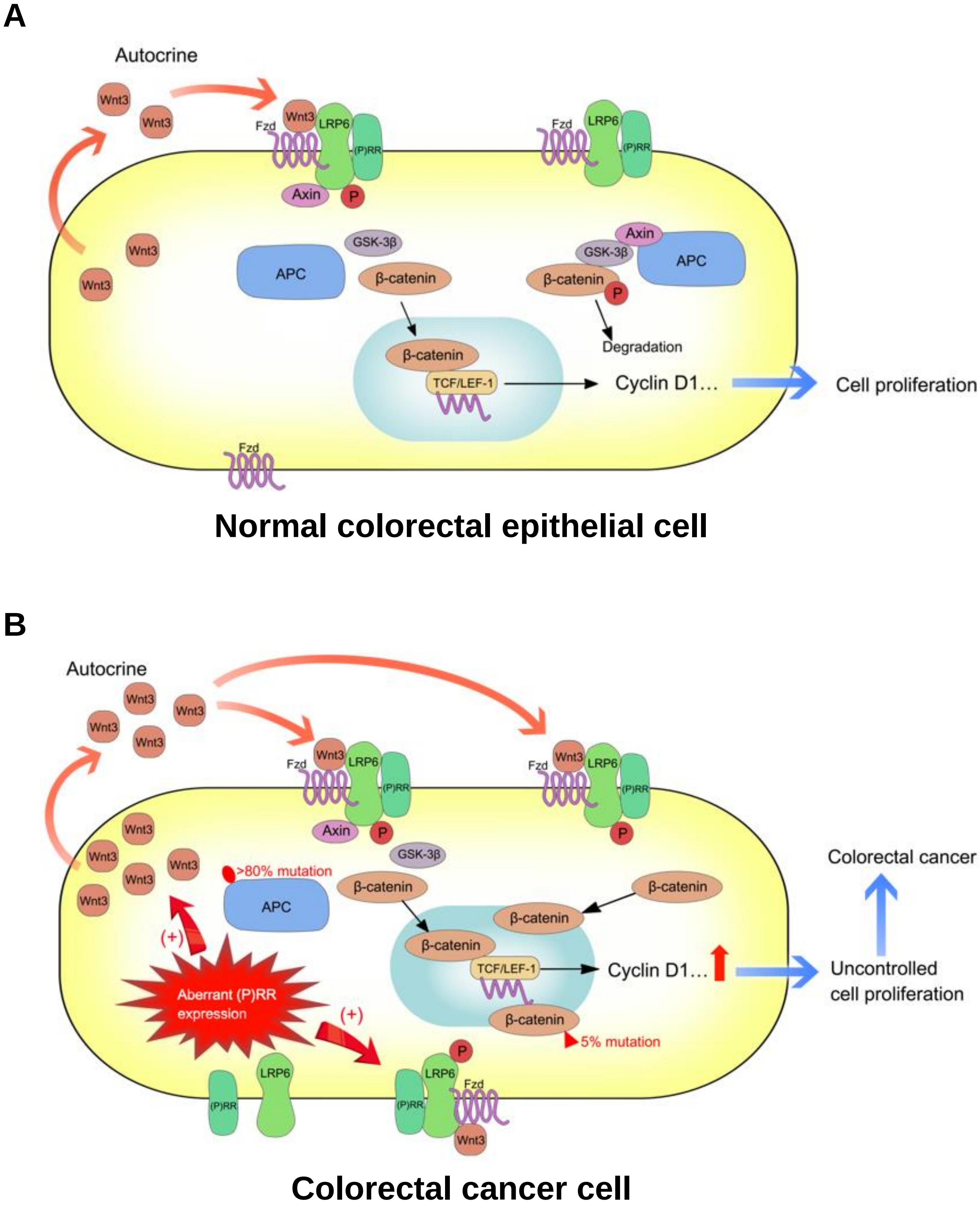
A
Autocrine
Wnt3
Fzd
LRP6
(P)RR
Axin
P
GSK-3β
APC
β-catenin
TCF/LEF-1
Degradation
Cyclin D1…
Cell proliferation
Normal colorectal epithelial cell
B
Autocrine
Wnt3
Fzd
LRP6
(P)RR
Axin
P
GSK-3β
>80% mutation
APC
β-catenin
(+)
Aberrant (P)RR expression
TCF/LEF-1
Cyclin D1…
5% mutation
Colorectal cancer
Uncontrolled cell proliferation
Colorectal cancer cell

Supplementary Figure S4. Schematic diagram summarizing the roles of (P)RR in promoting colorectal cancer through the Wnt/β-catenin signaling pathway. (**A**) In normal colorectal epithelial cells, the binding of endogenously secreted Wnt3 to its 'membrane receptor complex' keeps Wnt/β-catenin signaling at a normal level and thus plays a role in cell proliferation. (**B**) In colorectal cancer cells, by increasing endogenous Wnt3 and LRP6 protein levels, aberrant (P)RR expression excessively activates Wnt/β-catenin signaling and further promotes the disease, despite constitutive pathway activating mutations.
